# Microwave Radar Imaging of Heterogeneous Breast Tissue Integrating A Priori Information

**DOI:** 10.1155/2014/943549

**Published:** 2014-11-11

**Authors:** Jochen Moll, Thomas N. Kelly, Dallan Byrne, Mantalena Sarafianou, Viktor Krozer, Ian J. Craddock

**Affiliations:** ^1^Department of Physics, Goethe University of Frankfurt, Max-von-Laue-Straße 1, 60438 Frankfurt am Main, Germany; ^2^Centre of Communications and Research, University of Bristol, Bristol BS8 1UB, UK

## Abstract

Conventional radar-based image reconstruction techniques fail when they are applied to heterogeneous breast tissue, since the underlying in-breast relative permittivity is unknown or assumed to be constant. This results in a systematic error during the process of image formation. A recent trend in microwave biomedical imaging is to extract the relative permittivity from the object under test to improve the image reconstruction quality and thereby to enhance the diagnostic assessment. In this paper, we present a novel radar-based methodology for microwave breast cancer detection in heterogeneous breast tissue integrating a 3D map of relative permittivity as a priori information. This leads to a novel image reconstruction formulation where the delay-and-sum focusing takes place in time rather than range domain. Results are shown for a heterogeneous dense (class-4) and a scattered fibroglandular (class-2) numerical breast phantom using Bristol's 31-element array configuration.

## 1. Introduction

Breast cancer is one of the most prominent types of cancer in women with about 421,000 new cases being diagnosed in the European Union each year [1]. It is the most commonly diagnosed cancer and the leading cause of cancer death in women worldwide [2]. Thanks to national screening programs, a steady decline in breast cancer mortality can be observed [3]. Despite being the golden standard, X-ray mammography has severe limitations especially for screening dense breasts [4]. Adding to that the fact that still one in eight women suffers breast cancer, there is a need for new detection methods that will improve breast cancer diagnostics and treatment.

Microwave imaging (MWI) is an emerging imaging modality since the radiation is of low-power and nonionizing and provides complementary information to that obtained from X-ray mammography. A dielectric contrast at microwave frequencies between healthy and malignant breast tissue has been measured on a clinical basis [5], although this contrast is a function of the heterogeneity of human breast tissue and becomes smaller when the breast consists mainly of fibroglandular tissue.

In recent years, researchers improved the microwave approach for breast assessment by means of hardware innovations and new image reconstruction techniques [6]. Since microwaves at typical power densities are harmless for humans [7, 8], they can be employed not only for single diagnostic measurements but also for monitoring purposes. This has been demonstrated in [9] by controlling the healing process of neoadjuvant chemotherapy, where the dielectric tissue properties change over time. Another recent patient study has been reported in [10] employing a monostatic radar-based imaging system.

This work is motivated by the observation that conventional radar-based beamforming methodologies provide limited imaging capabilities in dielectrically heterogeneous imaging scenarios. A severe problem for many imaging algorithms related to microwave breast cancer detection (and similar to ground-penetrating radar) is given by the fact that the dielectric properties of the heterogeneous medium are not known a priori. Hence, the propagation velocity of the microwave signals is an unknown parameter for the imaging algorithm. This leads to the well-known effect that the signal energy does not coherently add up at the location of the scatterer so that its localization is affected by errors. In order to provide this information, researchers started to estimate the average static in-breast relative permittivity using either time of flight measurements [11] or transmission measurements through the breast [12]. An alternative solution is given by a time-domain inverse scattering technique that estimates the spatially averaged frequency-dependent dielectric properties of breast tissue [13].

In this paper, we will introduce a novel radar-based image reconstruction technique that goes a step further. Assuming a 3D permittivity model as prior information, we were able to identify a tumor in heterogeneous dense breast tissue, where conventional delay-and-sum imaging employing a constant relative permittivity fails. Throughout this paper, we postulate that the calibration is optimal in the sense that the dominant reflections from the skin and antenna coupling artifacts can be eliminated. This is a reasonable guess when a differential imaging is performed using contrast agents such as carbon nanotubes [14, 15] and bacterial microbots [16]. Such a differential contrast imaging process ensures an optimal calibration performing an initial scan of the breast in a first step. Then, a contrast agent is applied, which modifies the dielectric properties of the tumour target. After a second scan, the residual signals from both scans are applied to the imaging method, where the initial scan acts as the calibration signal which mitigates the effects of skin and healthy tissue scattering. Alternatively, rotational subtraction can be employed for differential imaging as demonstrated in [17].

A differential imaging method that exploits the variations in the propagation velocity can also be found in the context of guided-wave based structural health monitoring [18]. Here, the group velocity of the elastic waves changes as a function of the thickness of the structure and, in case of a fiber-reinforced material, as a function of propagation direction. For an accurate damage localization, especially in composite structures, the directional group velocity should be integrated into the image reconstruction formulation. A related application is acoustic emission testing, where the source location of an acoustic event can be identified with a higher accuracy as soon as a priori information about the anisotropic structure is available [19]. Source localization is also a fundamental problem in seismology, where the survey region is often geologically complicated and anisotropic. A recent review by Chouet and Matoza [20] discusses a variety of seismic source localization methodologies.

The remainder of this paper is organized in the following way.  Section 2 presents the novel imaging technique for general heterogeneous breast tissue that contains the solution for homogeneous dielectric tissue as a special case. Besides the mathematical theory, the numerical implementation will be described. After that, Section 3 presents not only the system model, but also several MWI results and a quantitative comparison. This section contains a detailed analysis when the permittivity map is affected by errors, that is, smoothing the permittivity map by a nonlinear 3D median filter and assigning a constant permittivity offset while keeping the anatomical complexity. Conclusions are drawn in Section 4 along with aspects of future work.

## 2. Radar-Based Image Processing Integrating A Priori Information

### 2.1. Mathematical Description

The first implementation of confocal microwave imaging can be found in [21]. Since then many image reconstruction techniques have been developed for radar-based MWI such as the robust Capon beamformer [22] or the coherence factor method [23]. All methodologies share the assumption of an average relative permittivity ${\bar{ɛ}}_{r}$ and hence an average wave velocity $\hat{c}=c/\sqrt{{\bar{ɛ}}_{r}}$ in the propagating medium. This allows a linear transformation of the time-domain signals *r*(*t*) to the range domain $r(s=\hat{c}t)$ which is used subsequently to form the 3D image.

In the following, we first consider conventional delay-and-sum (DAS) imaging, presented, for example, in [23, 24], since a systematic extension of the DAS technique will be derived subsequently. The DAS method determines the bistatic distances as the sum of the distances from the transmitter ${\vec{x}}_{i}$ to the point in the breast volume ${\vec{x}}_{P}$ and from breast tissue voxel ${\vec{x}}_{P}$ to the receiver ${\vec{x}}_{j}$. The corresponding signal amplitude is interpolated from the range profiles *r*_*ij*_(*s*) and assigned to the point in the breast. This can be mathematically expressed as

(1) Ix→P=∑i=1NT∑j=1NRrij(s)2=∑i=1NT∑j=1NRrijx→i−x→P+x→j−x→P2.

**Figure 1:**
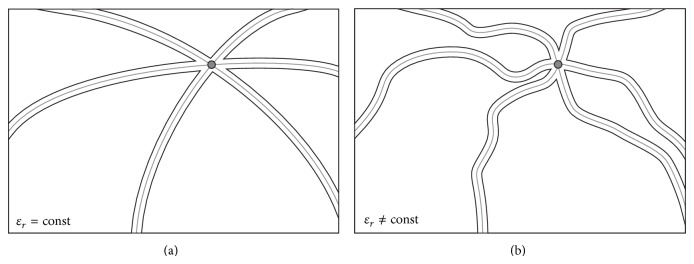
(a) Homogeneous DAS processing leads to ellipsoids that intersect and constructively interfere at the position of the scatterer (here three Tx-Rx pairs); (b) heterogeneous processing generates irregular surfaces crossing the location of the scatterer. Again three Tx-Rx pairs are considered.

Here, *N*_*T*_ denotes the number of transmitting antennas and *N*_*R*_ is the number of receiving antennas. Each transmitter-receiver pair produces an ellipsoid with the transmitter and the receiver at the focal points. The ellipsoids intersect and constructively interfere at the location of the scatterer as shown in Figure 1(a). When multiple transmitter-receiver contributions are considered, the position of the scatterer can be determined from the resulting maximum intensity. The DAS method benefits from a simple and efficient numerical implementation that can be greatly accelerated by modern computing platforms such as multicore CPU, graphics processing units (GPU), or FPGAs [25]. However, this procedure causes, as we have seen before, a systematic error since the heterogeneity of the female breast and thus the relative permittivity variation is generally not considered.

In this contribution, we propose the integration of a priori information about the breast's dielectric properties into the image processing formalism by means of a known three-dimensional permittivity model $P(\vec{x},f)$. Therefore, each voxel is not only defined by the three coordinates (*x*, *y*, *z*) but also by its additional relative permittivity *ɛ*_*r*_, that is, the static dielectric constant at the known center frequency of the excitation pulse. The special case of homogeneous dielectric tissue is automatically included as a special case. As a result of the spatially varying propagation velocity, the aforementioned linear transformation cannot be performed anymore. Hence, the image reconstruction needs to be performed in time domain rather than range domain. This leads to the time delay from the transmitting antenna to the voxel *t*_*iP*_ and from this voxel to the receiver *t*_*Pj*_:

(2) $$\begin{array}{c} t_{iP}=\frac{\left\|{\vec{x}}_{i}-{\vec{x}}_{P}\right\|}{c\left(f\left(\vec{x},f\right)\right)},\\ t_{Pj}=\frac{\left\|{\vec{x}}_{j}-{\vec{x}}_{P}\right\|}{c\left(f\left(\vec{x},f\right)\right)}. \end{array}$$

Note that the geometric distances are the same as before, but the underlying velocity depends on the location within the breast $\vec{x}$ and the frequency *f*. Hence, the intensity is given by

(3) $$\begin{array}{c} I\left({\vec{x}}_{P}\right)=\overset{N_{T}}{\underset{i=1}{\sum }}\overset{N_{R}}{\underset{j=1}{\sum }}{\left[r_{ij}\left(t\right)\right]}^{2}=\overset{N_{T}}{\underset{i=1}{\sum }}\overset{N_{R}}{\underset{j=1}{\sum }}{\left[r_{ij}\left(t_{iP}+t_{Pj}\right)\right]}^{2}. \end{array}$$

In contrast to the conventional DAS beamformer that produces an ellipsoid for each transmitter-receiver pair based on a constant propagation velocity, the general case of heterogeneous tissue generates irregularly shaped equipotential surfaces within the breast region as shown in Figure 1(b). Similarly, a summation over all transmitter-receiver combinations leads to an intensity distribution, where the highest intensity represents the tumour location. The shape of the surfaces highly depends on the anatomical complexity of the propagation medium and changes significantly from one Tx-Rx pair to another.

### 2.2. Numerical Implementation

**Figure 2:**
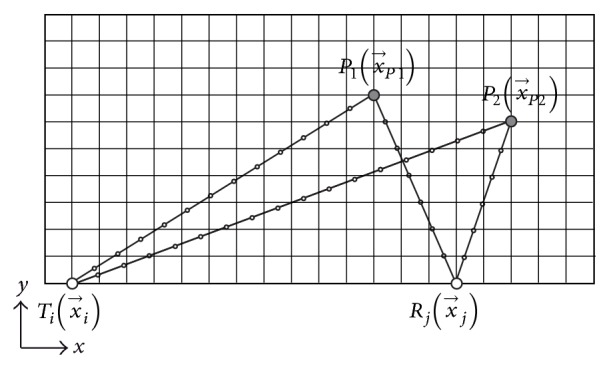
Bistatic geometry for time-delay estimation for the image reconstruction in heterogeneous breast tissue. The points on the straight generally do not coincide with the underlying voxel grid so that interpolation is required.

The numerical implementation starts with a spatial discretization of the volume of interest as shown in Figure 2. Each voxel (*k* = 1 ⋯ *N*_*V*_) is correspondingly defined by its coordinates (*x*_*k*_, *y*_*k*_, *z*_*k*_) and its relative permittivity *ɛ*_*k*_. The time delay for the wave to travel from the transmitter to the current voxel, here $P_{1}({\vec{x}}_{1})$ and $P_{2}({\vec{x}}_{2})$, and from the voxel to the receiver is calculated on an incremental basis. Therefore, *N*_*P*_ points need to be defined on a straight line between the known coordinates of the transmitter/receiver and the voxel. The corresponding velocity at each of the *N*_*P*_ points needs to be interpolated from the known 3D permittivity map. In the proposed implementation, a dedicated* interpns*-scheme is used that is a multidimensional simplex-based variant of linear interpolation [26]. It is used here, since it produces accurate and computationally efficient interpolation results. Finally, the total time delay used in (3) can be determined from incremental summation over all *N*_*P*_ points

(4) $$\begin{array}{c} t_{iP}=\overset{N_{P}}{\underset{m=1}{\sum }}\frac{\left\|{\vec{x}}_{m}-{\vec{x}}_{m-1}\right\|}{\left(1/2\right)\cdot \left[c\left(f\left({\vec{x}}_{m},f\right)\right)+c\left(f\left({\vec{x}}_{m-1},f\right)\right)\right]},\\ t_{Pj}=\overset{N_{P}}{\underset{n=1}{\sum }}\frac{\left\|{\vec{x}}_{n}-{\vec{x}}_{n-1}\right\|}{\left(1/2\right)\cdot \left[c\left(f\left({\vec{x}}_{n},f\right)\right)+c\left(f\left({\vec{x}}_{n-1},f\right)\right)\right]}. \end{array}$$

Note that the velocity in the denominator of this equation is the average velocity between two adjacent voxels on the straight line. In the following, the dispersive nature of the propagation velocity is not considered for simplicity reasons, so that the expression for the propagation velocity simplifies to $c(f({\vec{x}}_{n},f_{c}))$, where *f*_*c*_ is the carrier frequency of the excitation pulse.

The numerical approach described here is general and can be applied to any complicated breast tissue. This enables the application of the method to women with dense breasts and women where the breast consists mostly of fatty tissue. This technique relies on the hypothesis that a permittivity map of the breast will be available with the required accuracy and that effective contrast agents can be applied. A dielectric property map can potentially be obtained by a frequency shift in the reflection signals that varies as a function of the underlying tissue permittivity [27]. Assuming a dense network of microwave antennas, this approach might be a solution to measure 3D permittivity maps in the future on a patient specific basis without additional sensor technology. In addition, the above-mentioned methods [11–13] can be applied here as well. Alternatively, a permittivity model might be extracted from a secondary modality such as ultrasound, elastography, or thermoacoustic techniques. Therefore, clinical studies are required that compare the diagnostic content between microwaves and those three modalities.

## 3. Results

### 3.1. System Model

Finite difference time domain (FDTD) models [28] of the breast were developed to examine the performance of the proposed imaging technique. An FDTD model is created on the 012304 (heterogeneously dense) MRI-derived breast model, taken from the UWCEM breast phantom repository at the University of Wisconsin, Madison [29]. The intensity of each voxel in the MRI is estimated and mapped to appropriate dielectric properties in the resultant FDTD model [29]. All dielectric properties are based on studies by Lazebnik et al. [5, 30], whereas frequency-dependent dispersion is not considered. The exterior of the breast is modelled as a low-loss permittivity matching medium with *ϵ*_*R*_ = 9 and *σ*_*R*_ = 0.25 S/m.

**Figure 3:**
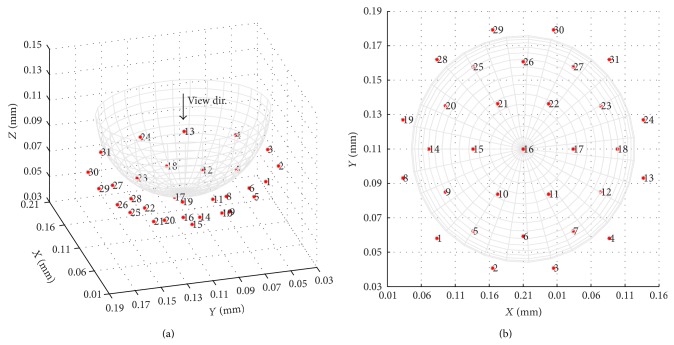
31-element multistatic array of point sources.

The FDTD grid resolution (*dx*, *dy*, *dz*) is 1 mm for each axis and the time step *dt* is defined as *dx*/2*c*, where *c* is the speed of light in a vacuum. The boundary of the FDTD domain is terminated using Mur absorbing boundary conditions [31]. Thirty-one point sources are arranged in Bristol's 31-element array configuration [32], as shown in Figure 3. Each element is excited in turn with a single cycle sinusoid with a raised cosine envelope and centre frequency of 2 GHz. Upon illumination of the breast, the remaining 30 multistatic receivers register any back scattering from the target. Due to reciprocity, this leads to a total number of 465 signals that are used for image reconstruction. A second FDTD data set is obtained, containing the internal breast tissues but omitting the tumour, to calibrate our initial scan data and to model a contrast-aided differential approach.

### 3.2. Image Reconstruction Examples

**Figure 4:**
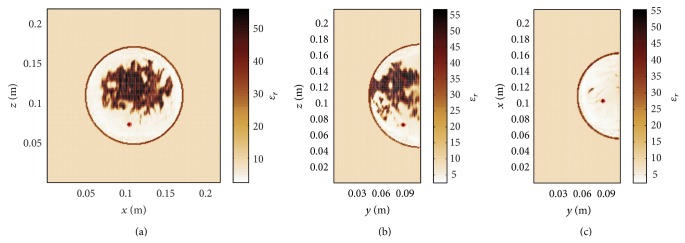
Heterogeneous dense numerical breast phantom with a tumour diameter of 4 mm at (0.104, 0.088, 0.074) m.

**Figure 5:**
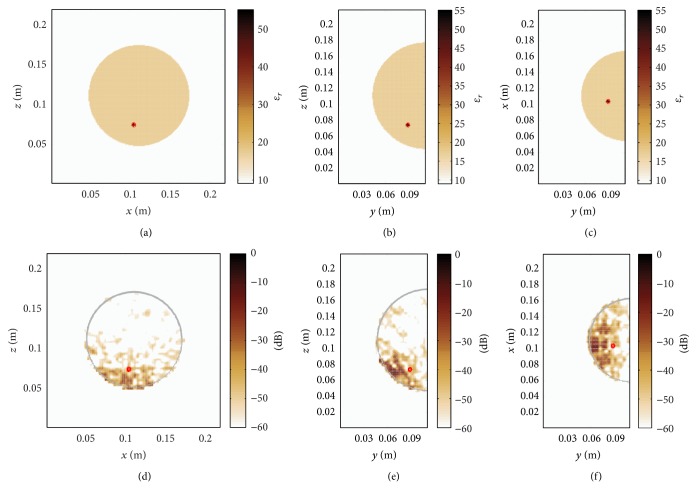
(a)–(c) Homogeneous map of relative permittivity, where the permittivity of the breast tissue is defined as the average in-breast permittivity of the heterogeneous breast shown in Figure 4. (d)–(f) Tumour localization fails when employing the average in-breast relative permittivity. The tumour position is indicated with a red circle.

Figure 4 illustrates the permittivity distribution in the heterogeneous dense breast. A tumour with a diameter of 4 mm is placed at the location (0.104, 0.088, 0.074) m. In a first step, the conventional DAS algorithm is used for image reconstruction. Here, the underlying average relative permittivity, that is, the static mean value of relative permittivity within the heterogeneous breast of *ɛ*_*r*_ ≈ 17.19, is determined from the in-breast area including the skin layer as shown in Figures 5(a)–5(c). The permittivity outside the breast is equal to *ϵ*_*R*_ = 9 so that only the permittivity variation inside the breast is subject to the present investigation. The average velocity is the best possible assumption that can be made for the conventional image processing. It can be seen from Figures 5(d)–5(f) that conventional DAS imaging is not able to resolve the tumour. From this observation it can be concluded that in the case of a heterogeneous breast and perfect calibration it is generally not possible to have a precise tumour localization assuming homogeneous conditions.

**Figure 6:**
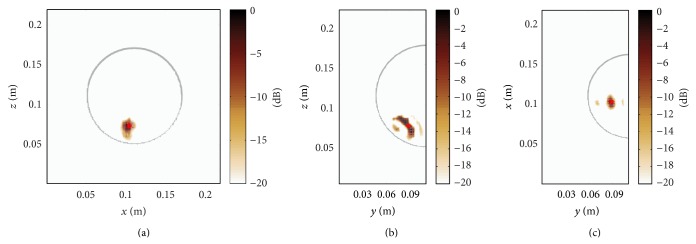
Time-domain DAS processing incorporating the true map of relative permittivity for accurate tumour localization.

Next, we consider the proposed heterogeneous time-domain image reconstruction technique, where the 3D permittivity map shown in Figure 4 has been exploited as prior information. As a result, Figure 6 demonstrates a clear focusing in all three dimensions. The signal to clutter ratio (SCR) that determines the ratio between the maximum and minimum detectable feature of the image is defined here as the intensity level on dB scale. A remarkable SCR of approximately 20 dB can be observed. This result proves that the time domain image reconstruction technique enables a precise tumour localization when the effects related to imperfect calibration are neglected.

**Figure 7:**
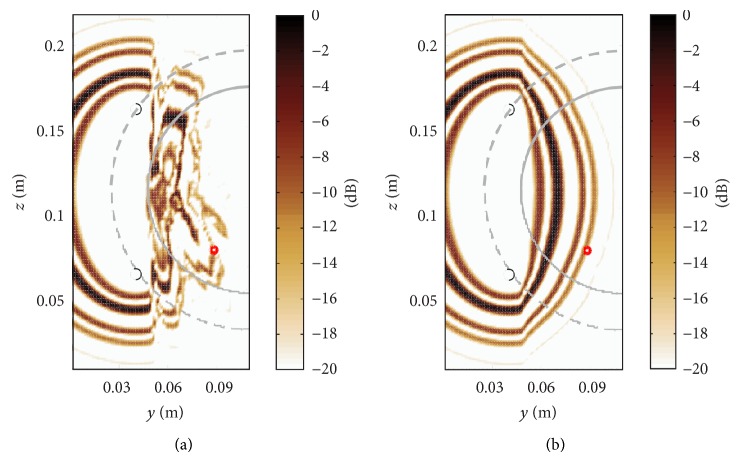
(a) Heterogeneous signal processing of Tx: 6 and Rx: 26 (black circles) leads to irregularly shaped equipotential surfaces within the breast region. Outside the hemisphere where the antennas are located (dashed line), the relative permittivity is constant which produces an ellipsoidal shape there. (b) Quasi-homogeneous processing of the same signals using the average relative permittivity of the in-breast tissue projects the large signal amplitudes not at the location of the tumour so that its localization fails. This example illustrates the systematic error that occurs through the homogeneity assumption.

In order to visualize the differences between the homogeneous and heterogeneous image processing, Figure 7 shows the contribution of a single transmitter-receiver pair. The heterogeneous processing is shown in Figure 7(a) that leads to an irregularly shaped equipotential surface within the breast region. Outside the hemisphere where the antennas are located, the relative permittivity is constant which produces, as expected, an ellipsoidal contour there. On the other hand, the homogeneous processing of the same signals using the average relative permittivity of the in-breast tissue projects the large signal amplitudes not at the location of the tumour, compare Figure 7(b), so that its localization fails. For an accurate localization of the tumour, it is therefore important to minimize the uncertainty related to the biological tissue by accounting for locally changing propagation velocity. In conclusion, this example illustrates the systematic error that occurs through the homogeneity assumption, which leads in this example to the fact that no meaningful tumour localization result can be obtained.

**Figure 8:**
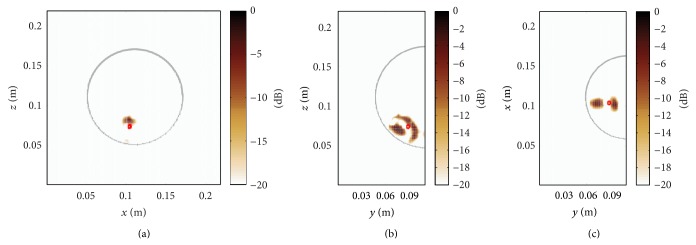
Keeping the anatomical complexity of the original breast geometry and employing a global bias in relative permittivity of (−1) leads to ambiguous tumour detection results in *y*-*z*-plane and *x*-*y*-plane.

**Figure 9:**
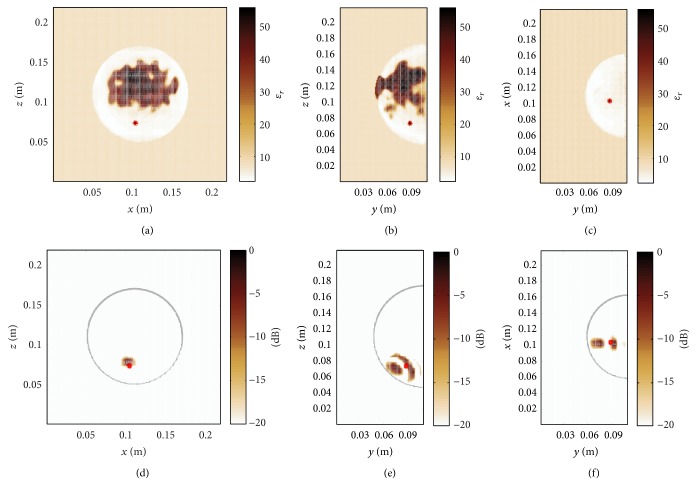
(a)–(c) Smoothed 3D map of relative permittivity using a 3D median filter of *N*_*o*_ = 7. (d)–(f) Additional peaks can be observed in *y*-*z*-plane and *x*-*y*-plane making the diagnostic result ambiguous.

Now, that we have found that a priori information about the dielectric properties is required for the detection of the tumour in heterogeneous dense breasts, the question is how good this model needs to be for accurate tumour imaging. To study this effect, we first consider a global bias in relative permittivity of (−1), leading to ambiguous tumour detection results shown in Figure 8. Further, we have used a nonlinear 3D median filter to smooth out anatomical details. The updated permittivity map is shown in Figures 9(a)–9(c). Note that due to the smoothing process the skin layer has been removed. The imaging result is presented in Figures 9(d)–9(f), where additional peaks can be observed in *y*-*z*-plane and *x*-*y*-plane making the diagnostic result ambiguous, too.

### 3.3. Quantitative Comparison Using Peak-to-Mean Ratio

Since a processing of the data with the conventional DAS method does not lead to a digital focusing, the following quantitative comparison is limited to the case of variants from the heterogeneous processing from Section 3.2. Therefore, the normalized peak-to-mean ratio is considered with the results shown in Table 1. The peak energy is the maximum pixel energy in a 3D spatial window around the known center point of the tumour, whereas the mean energy is the mean value of the energy in the remaining 3D domain outside the 3D spatial window. This ratio can be used to quantify the focusing performance where a higher value stands for better focusing quality. The values are normalized with respect to the highest peak-to-mean value.

Based on this definition and a radius of 6 mm for the spatial window, that is, three times the radius of the tumour, the proposed heterogeneous processing with the true permittivity map as a priori information produces the best overall results. Table 1 reveals a good focusing for the true permittivity with a median filter of *N*_*o*_ = 1. Slightly better focusing can be obtained in this case when the filter order is increased to *N*_*o*_ = 3. Further increasing the filter order and biasing the permittivity by (−1) reduce the focusing performance.

### 3.4. Comparison of Beamforming Techniques

**Figure 10:**
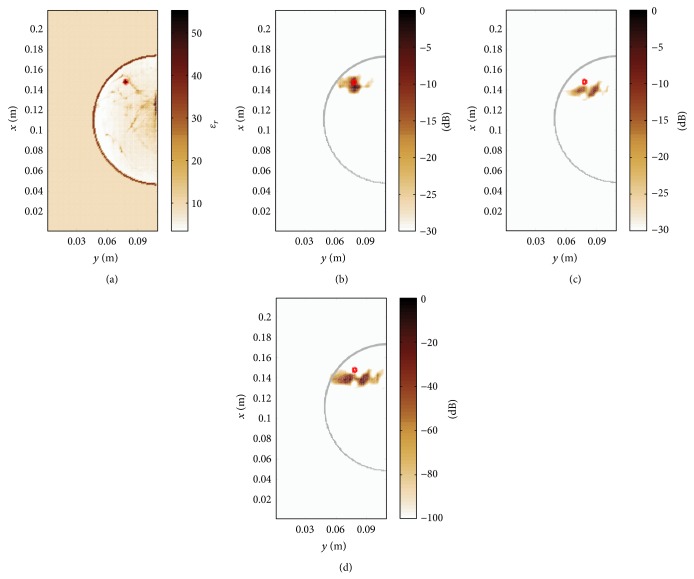
Class-2 numerical breast phantom (here *x*-*y*-plane) with a 4 mm tumour located at (0.148, 0.078, 0.097) m. (a) Map of relative permittivity, (b) image reconstruction result for the proposed method, (c) channel-ranked beamformer, and (d) coherence factor method.

In this section we will compare the performance of the proposed method with respect to more recent image reconstruction techniques, namely, the coherence factor method [33] and the channel-ranked beamformer [34].  Figure 10(a) shows the permittivity map of a class-2 numerical breast phantom with a reduced tissue complexity compared to the above-mentioned class-4 phantom in Figure 4. Figures 10(b)–10(d) show the imaging results of a planar slice for the proposed method, the channel-ranked beamformer, and the coherence factor method. The localization error, that is, the Euclidean distance between the known center of the tumour in 3D and the location of the maximum intensity, for the proposed method is 5.0 mm and increases for the channel-ranked beamformer and the coherence factor method to 13.1 mm. It can be concluded from this case study that the heterogeneous processing outperforms the two other reconstruction methods.

## 4. Conclusions

In this paper, a novel time-domain beamforming approach is proposed for radar-based tumour localization in dielectrically heterogeneous imaging scenarios. A special focus is on microwave breast cancer imaging in heterogeneous dense breast tissue using a multistatic radar with 31 transmitters and receivers. It was found that conventional delay-and-sum imaging, which assumes constant permittivity of the whole breast, is not able to resolve the tumour even in the case of perfect calibration and assuming the best possible average permittivity of the breast tissue. If we integrate the 3D permittivity map as prior information in the image reconstruction method, which can be obtained from coexisting imaging modalities, we are able to resolve the tumour with a high signal to clutter ratio of approximately 20 dB. Further, we biased and smoothed the 3D permittivity map which leads in both cases to ambiguous tumor localization results. From that observation it can be concluded that the integration of prior information for tumour localization is of major importance, especially for microwave imaging of heterogeneous dense breasts.

## Figures and Tables

**Table 1:** Evaluation of the reconstruction performance via normalized peak-to-mean ratio.

Description	Normalized peak-to-mean ratio
${\tilde{x}}_{N_{o}=1}$	0.939
${\tilde{x}}_{N_{o}=3}$	1
${\tilde{x}}_{N_{o}=5}$	0.775
${\tilde{x}}_{N_{o}=7}$	0.739
${\tilde{x}}_{N_{o}=9}$	0.762
${\tilde{x}}_{N_{o}=1,B=+1}$	0.529
